# Supplementary material for: Pre-injury activity predicts outcomes following distal radius fractures in patients age 60 and older
Source: PLoS One. 2020 May 20;15(5):e0232684. doi: 10.1371/journal.pone.0232684 (PMC7239474; doi:10.1371/journal.pone.0232684)
Supplement: S1 File — (DOCX) [file pone.0232684.s001.docx]

**S2 File. List of Intuitional Review Boards and Ethics Committees involved in WRIST.**

Carolinas Health Care Institutional Review Board

Copernicus Group

Duke Medicine Institutional Review Board for Clinical Investigators

Harvard Medical School Committee on Clinical Investigations

HealthPartners Research Foundation Office of Research Subjects

Feinstein Institute for Medical Research Human Research Protection Program

Fraser Health Research Ethics Board

Johns Hopkins Medicine Office of Human Subjects Research Institutional Review Boards

Mayo Clinic Institutional Review Boards

The MetroHealth System Intuitional Review Board

National Healthcare Group Domain Specific Review Board

Partners Human Research Committee
Presbyterian Healthcare Institutional Review Board

University of Connecticut Health Center Human Subjects Protection Office

University of Manitoba Office of Research Ethics

University of Michigan Medical School Intuitional Review Board

University of Oklahoma Health Sciences Center Institutional Review Board

University of Pennsylvania Office of Regulatory Affairs

University of Pittsburgh Institutional Review Board

University of Rochester Research Subjects Review Board

University of Washington Human Subjects Division, Institutional Review Board

University of Western Ontario Office of Research Ethics

Wake Forest University Office of Research, Institutional Review Board
