# Supplementary material for: Pre-injury activity predicts outcomes following distal radius fractures in patients age 60 and older
Source: PLoS One. 2020 May 20;15(5):e0232684. doi: 10.1371/journal.pone.0232684 (PMC7239474; doi:10.1371/journal.pone.0232684)
Supplement: Attachment — Submitted filename: response to reviewers.docx [file pone.0232684.s003.docx]

PONE-D-20-03237: Pre-Injury Activity Predicts Outcomes Following Distal Radius Fractures in Patients Age 60 and Older

**Journal Requirements:**

1. **Please ensure that your manuscript meets PLOS ONE's style requirements, including those for file naming.**

**Response:** We have revised the style to meet PLOS ONE requirements.

2. **Please provide additional details regarding participant consent. In the ethics statement in the Methods and online submission information, please ensure that you have specified (1) whether consent was suitably informed and (2) what type you obtained (for instance, written or verbal). If the need for consent was waived by the ethics committee, please include this information.**

**Response:** We have made this addition.

**Lines 195-196**: “Written informed consent was obtained from all WRIST participants.”

3. **Thank you for your ethics statement: "The WRIST protocol was approved by institutional review boards at all sites." Please amend your current ethics statement to include the full name of the ethics committee/institutional review board(s) that approved your specific study.**

**Response:** Because there are 24 sites in WRIST we have made the list of ethics committees and institutional review board available in a supplemental file.

S2 File. List of Intuitional Review Boards and Ethics Committees involved in WRIST.

4. **Once you have amended this/these statement(s) in the Methods section of the manuscript, please add the same text to the “Ethics Statement” field of the submission form (via “Edit Submission”).**

**Response:** this has been done

5. **In your Data Availability statement, you have not specified where the minimal data set underlying the results described in your manuscript can be found. PLOS defines a study's minimal data set as the underlying data used to reach the conclusions drawn in the manuscript and any additional data required to replicate the reported study findings in their entirety. Upon re-submitting your revised manuscript, please upload your study’s minimal underlying data set as either Supporting Information files or to a stable, public repository and include the relevant URLs, DOIs, or accession numbers within your revised cover letter.**

**Response:** this has been done

6. **One of the noted authors is a group or consortium [WRIST Group]. In addition to naming the author group, please list the individual authors and affiliations within this group in the acknowledgments section of your manuscript. Please also indicate clearly a lead author for this group along with a contact email address.**

**Response:** We have moved the list of WRIST contributors to the acknowledgment section.

**Reviewer #1:**

1. **Abstract: Please make clear if the stratification into highly and less-active groups took place before randomization or before analysis.**

**Response:** The stratification took place before the analysis. This has been added, line 97.

2. **Methods: Any other instability criteria?**

**Response:** These are the recommendations from the American Academy of Orthopedic Surgeons on the management of distal radius fractures and thus were used for the study.

3. **Results: I suggest to start this part with a positive sentence.**

**Response:** This has been changed, lines 236-237.

4. **From the data (especially the figures), I get the impression that highly active patients start high in the scores and end up high. However, the difference (delta) between 6 weeks and 12 months seem independent of the activity level and the treatment modality. All improved about the same %. Please comment on this topic.**

**Response:**

We have clarified this point.

Lines 281-285: “Although the change is similar, greater patient-reported outcomes *earlier* in the recovery process among highly-active patients over age 60, especially those who undergo VLPS (the most commonly used surgical treatment) is an important consideration during consultation with these patients to help them make a decision for or against surgery.”

5. **Patients after an operation usually get hand therapy. What kind of hand therapy did the enrolled patients receive?**

**Response:** We did not standardize hand therapy. Participants received the standard of care therapy regimen at their treating institution.

6. **Discussion: I expect some limitations due to multicentricity and enrollment of American, Canadian, and Singapore patients.**

**Response:** 99% of patients were from Canadian and American sites.

**Reviewer #2:**

1. **The claim of this paper is to examine patient-reported and functional outcomes in highly and less-active older participants following distal radius fracture. Additionally the authors assessed the relationship between pre-injury level of physical activity and postoperative outcomes after casting, volar plating system, percutaneous pinning and external fixator placement. How significant is for the traumatology to know the pre-injury level of physical activity if the surgeon choose the percutaneous pinning or the external fixator methods?**

**Response:** Our study demonstrates that traumatologists should be aware that highly-active patients who undergo percutaneous pinning have greater MHQ composite scores (including satisfaction and activities of daily living) compared to less-active patients; however, both percutaneous pinning and external fixation have very specific roles in the management of distal radius fractures (open fractures and significant comminution) related to pin site care, risk of infection, and non-rigid fixation. Regarding fixation and treatment methods, pre-injury level of activity should be used as a guide to help surgeons caring for these older patients. Despite chronological age, highly-active patients who wish to return to their normal activities in the shortest period of time should have the option to undergo surgery, preferably with volar locking plate.

**In the current literature there are main reasons in operating elder patient with isolated radius fractures. On the one hand they have bad soft tissue conditions on the other hand you have to avoid a long anesthesia period. The claims of the study are not properly placed in this context.**

**Response:** Although many of the patients likely have osteoporotic bone, all patients had closed distal radius fractures. Despite the swelling and soft tissue injury, wound complications following distal radius fractures are fairly low; Sirnio et al reported an ~0.8% culture-positive wound infection among 881 distal radius fracture patients who underwent volar locking plate [1]. Percutaneous pinning and external fixation pin site infections occurred in about 25% of participants [2].

Regional block is often the anesthesia of choice when fixing distal radius fractures; this obviates the medical risks associated with general anesthesia in this older group. Weighing the risks of wound complications, anesthesia and duration of procedure, treatment of highly-active older patients with distal radius reduction and volar plating is a reasonable option.

2. **The date and analyses support the claims but in their conclusion the authors are saying casting is a suitable treatment for less active patients based on comparable patient-reported outcomes. But this decision making has to set in a context related to soft tissue damage or other contraindication for an operative treatment of distal radius fractures.**

**Response:** Although the force required to cause the fracture would also cause significant soft tissue contusion, we did not have any open fractures in this series. The current study includes patients with closed distal radius fractures only; open wounds or substantial soft tissue compromise that necessitated surgery was not encountered. Despite edema, swelling, and soft tissue damage, few patients suffer wound healing complications following ORIF DRF with volar plating.

3. **Line 58: Volare plates cannot be used in all elderly patients. The above conclusion applies only to a selective patient population**

**Response:** Agreed. We have clarified this.

**Lines 106-108: “**This has been changed to, “Because of the greater PROs, the early mobility and lower risk of hardware infection reported in the literature, volar plating is preferable to other treatments for highly-active patients who request and meet indications for surgery.”

4. **Line 70: Casting is reserved for low-demand, less-active patients…. There are also active patients who simply do not want surgery and therefore also qualify for casting treatments.**

**Response:** We have edited this.

**Lines 121-124**: “Casting is reserved for low-demand, less-active patients or those patients who do not wish to undergo surgery. Surgical reduction and fixation, typically with a volar locking plate system (VLPS) is utilized in high-demand, active patients.”

5. **Line 99: The instability criteria listed are not complete. If this is wanted or there were no unstable radius fractures with termination of the palmar / dorsal joint lip, termination of the styloid ulnar process near the base, radioulnar dissociation, dorsal tilt of the peripheral fragment > 20 °, palmar tilt of the peripheral fragment > 20 °.**

**Response:** The criteria listed in the manuscript was used for the trial and thus were reported.

6. **Unfortunately, no tables were included in the manuscript for the reviewer. The table titles could only be read under numbers 358-363.**

**Response:** Tables are now included in the manuscript.

7. **Line 228/229: I think in the discussion a gradation of the criteria regarding the therapy algorithm should be worked out, whereby the preoperative activity level is weighted less than the fracture pattern**

**Response:** We agree the fracture pattern should be the most important factor; however, often times elderly patients who otherwise meet criteria for surgical intervention based on the previously listed criteria are not considered surgical candidates based on chronological age. The purpose of the study is to expand the way we think about suitability for wrist surgery among the elderly.

8. **Line 242/243: If you are already performing an operative intervention, you should aim at least for an exercise-stable osteosynthesis in order to be able to carry out a functional after-treatment. Otherwise the operational risk of complications is too great.**

**Response:** Agreed, this is why we recommend volar plating over the other two methods of fixation. Fortunately, volar plating is more commonly used for fixation than some of the other methods.

9. **Line 256/257: Even if the risk of falls in active patients surely increases, supervision cannot be required as a prerequisite for their activities. That would cause costs to rise massively**

**Response:** Agreed, complete supervision of all elderly activities would be costly. We advocate continued vigilance in identification and treatment of osteopenia and osteoporosis as falls in the setting of bone mineral deficiency leads to these fragility fractures.

**References:**

1. Sirnio K, Flinkkila T, Vahakuopus M, Hurskainen A, Ohtonen P, Leppilahti J. Risk Factors for Complications After Volar Plate Fixation of Distal Radius Fractures. J Hand Surgery European Volume. 2019; 44: 456-461.
2. Chung KC, Malay S, Shauver MJ, Kim HM, for the WRIST group. Assessment of distal radius fracture complications among adults 60 years or older: A secondary analysis of the WRIST randomized clinical trial. JAMA Netw Open. 2(1):e187053, 2019.
