# Supplementary material for: Pre-injury activity predicts outcomes following distal radius fractures in patients age 60 and older
Source: PLoS One. 2020 May 20;15(5):e0232684. doi: 10.1371/journal.pone.0232684 (PMC7239474; doi:10.1371/journal.pone.0232684)
Supplement: Attachment — Submitted filename: WRIST LOA Reviewer Comments Part 2.docx [file pone.0232684.s004.docx]

Reviewer #3: Regarding the statistical methodology used in this manuscript, I have the following concerns:

1. **Once a covariate has been detected as significant, please explain the interest of unadjusted comparisons between groups.**

We appreciate your comment. We intended to use the unadjusted differences to describe the imbalance between the two groups. However, it is not necessary to report the unadjusted p-values.

The modified table 3 has no unadjusted p-values. Formal comparisons are done by the controlled p-values.

2. **In this manuscript, an effect size measure is mentioned as if there were only one possible index, but there are several choices. To begin with, it must be highlighted that all the effect sizes are standardized ones, in opposition to the raw ones. On the other hand, there are different possibilities for these standardized measures. I am not sure if this manuscript reports Cohen's d or another related effect size. This must be properly stated. On the other hand, Cohen's d has been proved to be a biased standardized effect size and several corrections are available.**

Thank you for pointing this out. The effect size was measured using the standardized mean difference adapted from a paper by Kotsis et. al (<https://www-sciencedirect-com.proxy.lib.umich.edu/science/article/pii/S0363502306010859>) in a similar clinical setting.

We calculated the standardized mean difference in response using the formula: (mean response in highly active group – mean response in less active group)/SD of the measurement in two groups. It is used to measure the magnitude of difference between the two groups, which is different from Cohen’s d. Initially the Kotsis et. al uses ranges small (0.2-0.4), medium (0.50-0.70) and large (>0.80) as cutoffs. In our scenario, the effect sizes can be negative when less active group have better response. However, the absolute number of effect size specifies the magnitude of the difference clinically and could follow the same ranges. This is present in the manuscript, lines 152-156.

3. **Moreover, regarding the effect sizes, I disagree with the idea of these measures are not applicable when a difference is lacking statistical significance. Precisely, the p-values are affected by the sample size while the standardized effect size measures are not. So, standardized effect sizes are a good guideline to assess the statistical power involved in a comparison. On the other hand, there are comparisons (eg table 3) with statistical significance where the effect size is not available.**

Thank you so much for your comment. We have added the effect sizes for non-significant parameters and unadjusted comparisons in tables 3 and 4. For table 3, if the estimators are not statistically different from 0, the effect sizes would be small. For table 4, the effect sizes examine the magnitude of the conditional mean difference at a specific time point. We used reference cell coding for time as a categorical variable (please find further explanation in point 5).

4. **Linear Mixed Models and GEE are different methodologies. The first one is not mentioned in the section of methodology but it is used to obtain the results in table 4. The election of each methodology must be properly explained.**

We apologize. There was a typo in the label for table 4. We corrected the label as the “GEE model results”. The GEE model was preferred compared to the linear mixed model for its flexibility and allowing complicated correlation structures. Moreover, it only needs the 1^st^ moment to be correctly specified rather than the whole likelihood. This has been updated in the manuscript, lines 163-168.

5. **Regarding table 4, I find weird to give one coefficient estimation of the model for each instant of measure. If the model considers time as a variable, there must be only one coefficient for it (considering the trend is linear), not one coefficient for each time value.**

In the GEE models (table 4), we did not assume time to be linear. We used reference cell coding for time as a categorical variable, since we only have 4 time points in the data.

The model for each patient outcome is specified as:

$\text{E(Response}_{ij})=\beta_{0}+\beta_{1}I\left( \text{patient i is highly active } \right)+ \beta_{2}I\left( \text{patient i is highly active} \right)\times I( \text{time}_{ij}=\text{3 months})$ + $\beta_{3}I\left( \text{patient i is highly active} \right)\times I\left( \text{time}_{ij}\text{ = 6 months} \right)+\beta_{4}I\left( \text{patient is highly active} \right)\times I\left( \text{time}_{ij}\text{ = 12 months} \right)+\text{demographics}_{i}$

We have updated table 4 to better describe the model. The estimated mean differences are $\beta_{1}$(6 weeks), $\beta_{1}+\beta_{2}$ (3 months), $\beta_{1}+\beta_{3}$(6 months) and $\beta_{1}+\beta_{4}$ (12 months) respectively.

Previously, we have reported p-values for $\beta_{1}-\beta_{4}$ in table 4. To better examine the group differences controlling for treatment received, time, within-patient correlation and other covariates, we have updated the p-values in table 4 with Wald test results testing $H_{a}:\beta_{1}\neq0$,$H_{a}: \beta_{1}+\beta_{2}\neq0$, $H_{a}: \beta_{1}+\beta_{3}\neq0$ and $H_{a}: \beta_{1}+\beta_{3}\neq0$ for time specific mean difference in response between highly active and less active groups.

6. **My view is that there is a high level of duplicity between the information given in tables and that which is given in the text.**

Thank you for your comment. The tables provide all the statistical findings, we wanted to highlight some clinically important statistical findings from the tables in the text. This has been minimized.

7**. Finally, decimal figures of p-values must be unified. APA's recommendation is to use three.**

Thank you for the suggestion. We have updated the tables 1-4 to unify 3 decimal figures for p-values.
